# Complications of high grade liver injuries: management and outcomewith focus on bile leaks

**DOI:** 10.1186/1757-7241-20-20

**Published:** 2012-03-23

**Authors:** Miklosh Bala, Samir Abu Gazalla, Mohammad Faroja, Allan I Bloom, Gideon Zamir, Avraham I Rivkind, Gidon Almogy

**Affiliations:** 1 Department of General Surgery and Trauma Unit, Hadassah-Hebrew University Medical Centre, Kiriat Hadassah, POB 12000, Jerusalem 91120, Israel; 2 Department of Interventional Radiology, Hadassah-Hebrew University Medical Centre, Kiriat Hadassah, POB 12000, Jerusalem 91120, Israel

**Keywords:** high grade liver trauma, complications, bile leak, ERCP, non operative management

## Abstract

**Background:**

Although liver injury scale does not predict need for surgical intervention, a high-grade complex liver injury should alert the physician to expect an increased risk of hepatic complications following trauma. The aim of the current study was to define hepatic related morbidity in patients sustaining high-grade hepatic injuries that could be safely managed non-operatively.

**Patients and methods:**

This is a retrospective study of patients with liver injury admitted to Hadassah-Hebrew University Medical Centre over a 10-year period. Grade 3-5 injuries were considered to be high grade. Collected data included the number and types of liver-related complications. Interventions which were required for these complications in patients who survived longer than 24 hours were analysed.

**Results:**

Of 398 patients with liver trauma, 64 (16%) were found to have high-grade liver injuries. Mechanism of injury was blunt trauma in 43 cases, and penetrating in 21. Forty patients (62%) required operative treatment. Among survivors 22 patients (47.8%) developed liver-related complications which required additional interventional treatment. Bilomas and bile leaks were diagnosed in 16 cases post-injury. The diagnosis of bile leaks was suspected with abdominal CT scan, which revealed intraabdominal collections (n = 6), and ascites (n = 2). Three patients had continuous biliary leak from intraabdominal drains left after laparotomy. Nine patients required ERCP with biliary stent placement, and 2 required percutaneous transhepatic biliary drainage. ERCP failed in one case. Four angioembolizations (AE) were performed in 3 patients for rebleeding. Surgical treatment was found to be associated with higher complication rate. AE at admission was associated with a significantly higher rate of biliary complications. There were 24 deaths (37%), the majority from uncontrolled haemorrhage (18 patients). There were only 2 hepatic-related mortalities due to liver failure.

**Conclusions:**

A high complication rate following high-grade liver injuries should be anticipated. In patients with clinical evidence of biliary complications, CT scan is a useful diagnostic and therapeutic tool. AE, ERCP and temporary internal stenting, together with percutaneous drainage of intra-abdominal or intrahepatic bile collections, represents a safe and effective strategy for the management of complications following both blunt and penetrating hepatic trauma.

## Background

Treatment of traumatic liver injuries is based on patient physiology, mechanism and degree of injury, associated abdominal and extra-abdominal injuries and local expertise. Non-operative management has evolved into the treatment of choice for most patients with blunt liver injuries who are hemodynamically stable and success rates for non-operative management commonly are greater than 95% [1]. The rate of liver-related complications is low, and generally ranges from 0% to 7% [1-5]. However, the majority of patients in these studies suffered from low-grade liver injuries.

Liver-related complication rates in high-grade liver injury patients are 11-13% and can be predicted by the grade of liver injury and the volume of packed red blood cells transfused at 24 hours post-injury [4,6,7]. Mohr and colleagues studied complications related with angiographic embolization (AE) and found a morbidity rate of 58% for long-term survivors with blunt liver injury [8]. Bile leak, liver abscess, and ischemic necrosis of the liver and gallbladder were some of the more common complications. A quarter of the patients who were treated operatively developed complications such as liver abscess and bile leak that consequently required surgery. Mortality for this group of patients was 27% [8]. Carrillo described complications in up to 85% of patients with a high (≥4) Abbreviated Injury Score (AIS) in a series of 32 patients who were treated non-operatively [9].

The introduction of non-operative strategies as well as improvement of operative techniques and perioperative care for the management of blunt liver injury has successfully reduced liver-related mortality. Our current attention should be to reduce liver-related morbidity in order to optimize outcome. The goals of this study were to analyze the complication rate following high-grade liver injuries and assess the safety and efficacy of non-operative strategy in management of patients with different types of liver-related complications following liver trauma.

### Patients and methods

All patients with blunt and penetrating liver injury presenting to Hadassah-Hebrew University Medical Centre, Jerusalem, Israel, between January 2000 and December 2009 were included in the study. All charts containing intervention reports were reviewed retrospectively. Collected data included age, sex and mechanism of injury; Injury Severity Score (ISS), length of stay (LOS) and outcome. The grading of liver injury was based on contrast-enhanced computed tomography (CT) or laparotomy findings, according to the American Association for the Surgery of Trauma Organ Injury Scale for hepatic injuries [10]. Grade 3-5 liver injuries were considered high-grade and included in the study group. All liver related complications were analyzed, in terms of timing of appearance and mode of diagnosis and treatment. All deaths were reviewed for cause.

Initial treatment, resuscitation and investigation were carried out based on the guidelines of the Advanced Trauma Life Support. Patients were assigned to surgical or non-surgical treatment based on the mechanism of trauma and their hemodynamic status.

In hemodynamically stable patients, abdominal CT was performed and, if an arterial contrast extravasation was present, early AE was performed. The need for initial AE was not considered a bleeding complication, but part of our institutional treatment protocol for liver trauma. In patients with persistent hemodynamic instability an immediate laparotomy was performed. Multiple staged procedures with initial packing and non-anatomic hepatic debridement were done sequentially, when damage control mode was indicated. Hepatic artery AE was an adjunct to damage control mode, if clinically required or contrast extravasation was found on postoperative CT scan. Follow up CT was performed based on clinical and/or laboratory suspicion of biliary complications such as elevated temperature, right upper quadrant pain, abdominal distension, and melena.

"Liver-related" complications were defined as biliary (bile leak, biloma), delayed bleeding, infectious (hepatic and perihepatic abscesses), cholecystitis and liver failure. The definition of bile leak was based on the continuous drainage of bile through an intra-abdominal drain after surgery, or following percutaneous drainage of an intra/extra hepatic collection. Complications which appeared in victims who expired within 24 hours of admission were excluded.

A multidisciplinary approach was used in the treatment of liver related complications. It included AE, endoscopic retrograde cholangiopancreatography (ERCP) or percutaneous transhepatic biliary drainage (PTBD) and stenting of biliary ducts, and CT-guided drainage of hepatic or perihepatic abscess, or biliary collections. Antimicrobial therapy was initiated according to the results of drainage and blood cultures. Surgical interventions for treating hepatic complications were considered if minimally invasive management failed. When biliary leaks were treated by ERCP, a 7 or 10 French plastic stent was inserted with or without endoscopic sphincterotomy (ES). The ERCP was performed with a therapeutic duodenoscope (TJF 140R, Olympus Opticals Ltd. Tokyo, Japan). While performing biliary stenting, no attempt was made to place the stent across the injured duct. The stent was left in the distal ductal system to aid bile drainage. Post ERCP follow up was monitored by clinical assessment and by monitoring of bile output from the drains. Recovery was defined as the cessation of leakage or absence of radiological evidence of a biliary leak. Drains were removed once recovery had been achieved. Stent removal was planned 3-6 months after insertion. Follow up at the surgical in-patient clinic continued for up to 6 months after discharge.

### Statistical analysis

The data is expressed as mean ± standard deviation. All analyses were done with SPSS for Windows Version 15.0 (SPSS, Chicago, IL, USA). A *p *value of < 0.05 was considered statistically significant.

## Results

### Patient characteristics

There were 398 patients who presented with hepatic injuries, of which 64 (16%) had grade 3, 4, or 5 injuries, which make up the study group. Table 1 provides a breakdown of these high-grade injuries. The most common mechanisms of injury were motor vehicle crashes (n = 33, 52%), gunshot wounds (n = 12, 18.7%), and terror-related injuries caused by shrapnel (n = 7, 11%).

**Table 1:** Liver injuries by demographic, ISS, management, number of complications and mortality

	Grade 3	Grade 4	Grade 5	All	*P *value
	(n = 14)	(n = 34)	(n = 16)	(n = 64)	
Age, mean (± SD)	27.5 ± 16.8	23 ± 14.3	29 ± 16.1	24.8 ± 15.5	NS

Gender, male (%)	9 (64.3)	22 (64.7)	13 (81.3)	44 (68.8)	NS

ISS, mean (± SD)	33.8 ± 14.4	33.3 ± 13.6	36 ± 11.7	35.3 ± 13.4	NS

Operative management (%)	6 (43%)	19 (56%)	15 (94%)^#^	40 (62%)	0.008

Overall mortality (%)	3 (21%)	10 (29%)	11 (69%)	24 (37.5%)	0.01

Gender, male (%)	9 (64.3)	22 (64.7)	13 (81.3)	44 (68.8)	NS

ISS, mean (± SD)	33.8 ± 14.4	33.3 ± 13.6	36 ± 11.7	35.3 ± 13.4	NS

Operative management (%)	6 (43%)	19 (56%)	15 (94%)^#^	40 (62%)	0.008

Overall mortality (%)	3 (21%)	10 (29%)	11 (69%)	24 (37.5%)	0.01

ISS - injury severity score; NS - non significant difference; ^# ^- grade 5 data significantly different from grades 3 and 4.

There were 24 deaths (37.5%). Eighteen victims (75%) died on the day of admission from haemorrhagic shock. Four patients died from multiorgan failure on days 11 through 97. Compared with patients with grade 3 and 4 liver injury, patients with grade 5 injury had a significantly overall higher mortality rate (21%, 29%, and 69%, respectively, p < 0.01) (Table 1). There were two liver-related mortalities secondary to hepatic failure in patients with grades 4 and 5 injury.

### Liver-related complications

**Figure 1:**
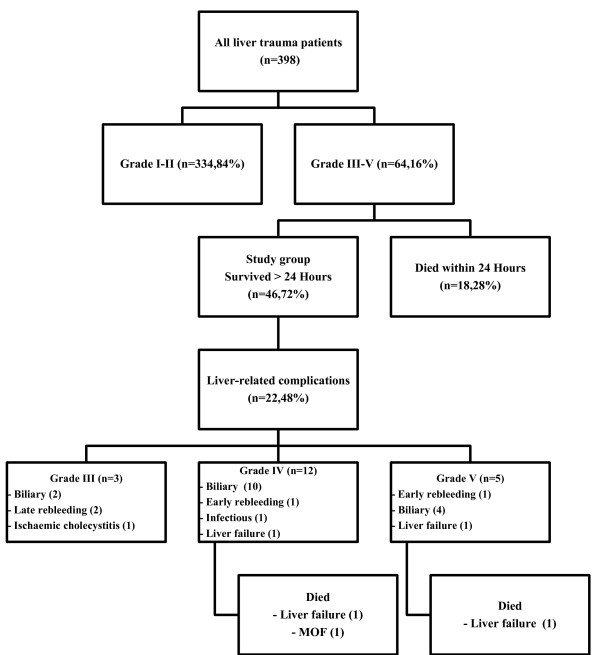
**Flow chart of liver trauma patients, liver-related complications and outcome**.

22 of the 46 patients (47.8%) who survived longer than 24 hours developed a total of 24 liver-related complications (Figure 1). Complications included bile leaks (n = 11), bilomas (n = 5), rebleeding episodes (n = 4), intrahepatic abscess (n = 1), acute cholecystitis (n = 1) and liver failure (n = 2). Three of the 11 patients (27%) with grade 3 injury developed 5 complications, 12 of the 28 patients (43%) with grade 4 injury developed 13 complications, and, 5 of 7 patients (71%) with grade 5 injury developed a total of 6 complications (*p *not significant).

### Biliary complications

**Figure 2:**
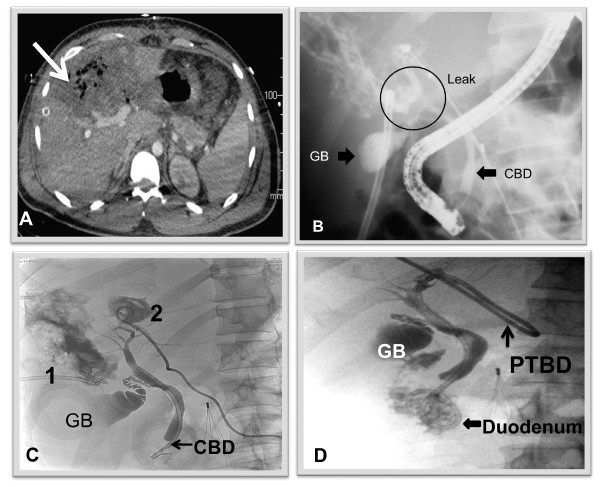
**A 25 year old male suffered a trans-abdominal GSW with grade 4 liver injury, duodenal and small bowel injuries**. On PID 12 intrahepatic abscess was found on CT scan (**A**, arrow). A bile leak developed following percutaneous drainage. An ERCP (**B**) was performed and showed a leak from both right and left hepatic ducts (GB - gallbladder, CBD - common bile duct, leak from both ducts is encircled). Following image (**C**) showed well-drained intrahepatic bilomas (percutaneous drains 1 and 2). PTBD was inserted instead of a percutaneous drainage (**D**). It was removed 4 months later when no contrast leak was found and the patient tolerated closure of the external part of the PTBD.

There were 11 patients (23.9%) with bile leaks following traumatic liver injury. The mechanisms of injury were motor vehicle accidents (n = 8) and penetrating injuries (n = 3). The diagnosis of a bile leak was suspected on abdominal CT scan, which revealed intra-abdominal collections (n = 6) and ascites (n = 2), and continuous biliary leak from intra-abdominal drains that were placed during laparotomy (n = 3). The mean time interval between injury and diagnosis of a bile leak was 8 days (range 5-19 days). In 7 of these patients (63.6%) ERCP was performed successfully and revealed bile leaks from branches of the right hepatic duct (n = 5), and both right and left hepatic ducts (n = 2). Two patients underwent ES with stent insertion and 5 patients had stent insertion alone. In all procedures a biliary stent (Amsterdam plastic stents 7 or 10 French) was placed distal to the site of the leak. In one patient repeat ERCP was performed for stent dislodgement. One patient with liver injury and duodenal repair was initially managed withPTBD after postoperative bile leak appeared on post-injury day (PID) 12. He had an ERCP on PID 25 in order to discontinue external biliary drainage (Figure 2). In one case the papilla could not be cannulated and surgical drainage was performed on PID 7. Three months later the patient underwent right hepatectomy for right hepatic duct disruption. There were no ERCP related complications. In 3 post-operative patients without major ductal injury, minimal bile leaks resolved spontaneously.

There were 5 bilomas and one intrahepatic abscess which were diagnosed by CT and treated by percutaneous drainage on mean PID 14 (range 10 to 28).

After endoscopic and percutaneous interventions bile leaks resolved in all patients at a mean of 26 days (range 3-68 days), as determined by cessation of biliary discharge from abdominal drains and by radiological evidence for the absorption of intra-abdominal fluid collections. Repeat cholangiograms were performed in 6 patients before stent or PTBD removal and revealed no radiographic evidence of bile leak and normal anatomy of bile ducts.

### Bleeding complications

**Figure 3:**
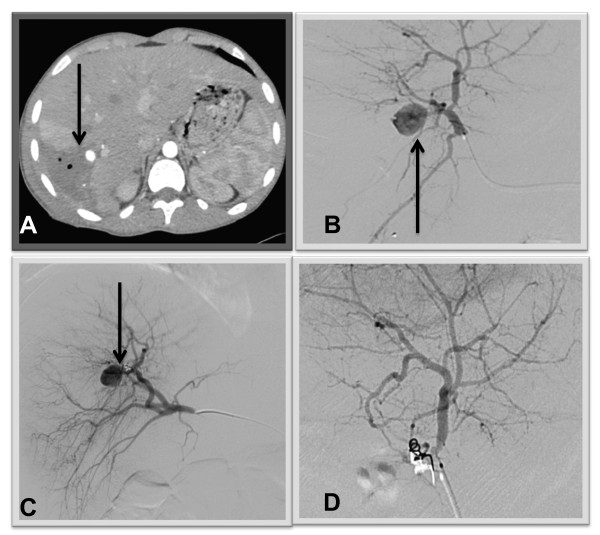
**16 year old male sustained a gunshot wound to the right upper abdomen**. He was managed non-operatively. Right hepatic artery pseudoaneurysm (PSA) was found on CT performed due to fever, billious discharge from the exit wound and pain on PID 16. Two days after successful AE he had an episode of external bleeding from the same bullet wound. Recurrent PSA was found on repeat angiography. Repeat AE was performed successfully. **A**: Contrast-enhanced CT scan obtained on PID 16 shows a newly developed, well-circumscribed PSA (arrow) within the perihepatic abscess containing air bubbles. **B**: On selective arteriogram, the PSA (arrow) is seen to arise from the right hepatic artery. **C**: Recurrent PSA found 2 days after previous AE. Coils are seen in the artery (arrow). **D**: Angiogram after successful AE.

Three patients (6.5%) developed four episodes of rebleeding, all successfully treated by AE. The source of bleeding in all episodes (100%) was the right hepatic artery. Two early rebleeding episodes appeared on PID 3 and 4 in patients with grade 4 and 5 liver injury. Two additional late rebleeding episodes occurred on PID 16 and 18 in one patient with a GSW and were related to infected biliary collections (Figure 3).

### Liver failure

Two patients died of liver failure in the postoperative period. One patient had grade 4 liver injury following blunt trauma. Right hepatectomy was performed following repacking. Relaparotomy revealed an ischemic liver remnant and he expired on PID 20. The second patient suffered from grade 5 liver injury caused by a GSW and had repacking 3 times. On PID 10 the patient developed liver necrosis with coagulopathy and hyperbilirubinemia and expired on PID 11 due to uncontrollable liver failure and coagulopathy. Both patients suffered from prolonged shock following injury and from severe hemorrhagic shock requiring massive transfusions and hemodynamic support.

### Effect of management strategy on complication rate

In order to analyze whether different factors could influence the development of liver-related complications, the patients were divided into 3 groups (Table 2). Univariate analysis showed that biliary complications (bile leaks and bilomas) among survivors for longer than 24 hours were more common in the operative group compared with the non-operative group, but the difference did not reach statistical significance (p = 0.184). Multivariate regression logistic analysis was performed to identify predictors of posttraumatic liver complications. Age, sex, mechanism of injury, grade of liver injury, operative management and the need for AE, were entered into a regression model which showed that operative management was found to be a predictor of a higher overall complication rate (adjusted OR = 4.286, 95% confidence interval 1.246 to 14.735, p = 0.02). None of the parameters was found to be a predictor of biliary complications.

**Table 2:** Analysis of factors influenced development of complications, including biliary complications in the study group

	Complications	No complications	Biliary complications	*P *value
	(n = 22)	(n = 24)	(n = 15)	
Male	17	17	13	NS

Age (years)	23.7 ± 11.9	20.6 ± 15	22.5 ± 12.2	NS

ISS (mean ± SD)	36 ± 14	32.6 ± 15	35 ± 15	NS

Grade of liver injury (mean ± SD)	4 ± 0.6	3.8 ± 0.6	4 ± 0.75	NS

Angioembolization (%)	6 (27.2)	5 (20.8)	5 (33.3)	NS

OR (%)	15 (68.2)*	8 (33.3)	9 (60)	0.038

Penetrating injury (%)	7 (31.8)	5 (20.8)	5 (33.3)	NS

OR - operative group; ISS - Injury severity score; NS - Differences not significant; * - p < 0.05 - complication rate was higher in OR patients.

Seven patients (29.2%) in the non-operative group and 4 patients (10%) in the operative group underwent AE upon admission. Patients who underwent AE upon admission were significantly more likely to develop biliary complications compared to patients who did not undergo AE (7 of 11 vs. 9 of 35, respectively, p = 0.032). Of the 11 patients with bile leaks, 10 (90.9%) developed following either operative management or AE. One patient developed acute ischemic cholecystitis 19 days after selective embolization of the right hepatic artery. He was initially managed with cholecystostomy and underwent an uneventful cholecystectomy 2 months later.

### Follow up

Thirteen of the 16 patients (81.3%) who developed biliary complications were available for follow up. All 13 patients remained asymptomatic after a median follow up of 7.5 months (range 3-24) from discharge. All post ERCP stents and PTBD were removed in the outpatient clinic. One patient had a cholecystectomy 2 months after discharge (mentioned above) and one had an incisional hernia repair 20 months following laparotomy.

## Discussion

The shift towards non-operative management of liver injuries has resulted in a lower mortality rate, but still a significant percentage of complications [3]. We report on complications and the required interventions for the management of complex liver injuries. Overall, 5% of patients with liver injuries developed complications, all in patients with grade 3 to 5 injuries. The complication rate among survivors for more than 24 hours was 47.8% (22 of 46 patients). 77% of patients who developed complications had either grade 4 or grade 5 liver injuries. The low incidence of complications with lower grade injuries, including grade 3 has been corroborated by others [7,9].

The complication rate in liver trauma patients who were treated non-operatively was lower compared with patients who underwent surgical hemostasis (27.2% vs. 50%, respectively).

According to our findings, operative management was a predictor of a higher overall complication rate. The combination of non-favorable patient physiology, surgical hemostasis, and high-grade liver injury are also related to the higher number of complications in other series [11].

Patients who were treated operatively and those who underwent AE tended to have higher rates of biliary complications and required additional interventions. The combination of traumatic injury and ischemia caused by embolization could certainly predispose to hepatic necrosis and biliary complications [12].

The prevalence of delayed haemorrhage following non-operative management of blunt liver injury ranges from 1.7 to 5.9% [9,13]. The mechanism of delayed hemorrhage may be related to an expanding injury or to a PSAinduced by a biloma which eventually causes an expanding hematoma and free rupture into the peritoneal cavity. Early bleeding episodes are attributed directly to the traumatic insult, while late hemorrhage is probably related to infectious hepatic complications. The patient with a late bleeding episode (PID 16) in our series was also diagnosed with an infected biloma prior to bleeding. Urgent AE was the modality used to treat rebleeding in the 3 patients with PSAs that were found on CT.

Following blunt hepatic trauma, biliary complications have been reported in 2.8 to 7.4% of patients [7,9]. The presence of a biloma on CT is suggested by the progressive growth of a well-circumscribed, low-attenuation intra-parenchymal or peri-hepatic collection [14]. We performed repeat CT scans based on the appearance of clinical signs and symptoms of liver-related complications such as right upper quadrant pain, jaundice, fever, or melena. Our data show that the optimal time period from injury to repeat imaging studies for high-grade liver injuries seems to be 7 to 10 days, the mean time for the appearance of complications. Almost three-fourths (72.7%) of bile leaks in our series were diagnosed when a CT showed a collection or intra-abdominal fluid which were suspicious for a bile leak. Diagnosis was confirmed by a diagnostic or therapeutic percutaneous drainage procedure. For asymptomatic patients with low-grade liver injuries follow-up CT is generally unnecessary [15].

ERCP with stenting and percutaneous drainage procedures formed the mainstay of therapy used in the treatment of bile leaks for patients with liver trauma. As often seen in elective liver resections, most peripheral biliary leaks will seal without intervention. Marks and co-workers suggested that stenting, rather than spincterotomy was more effective in resolving biliary leaks [16]. Our experience has shown that continuous high output biliary drainage should be managed by ERCP and stenting to allow for healing. Early ERCP, i.e. within 24 hours, is essential in the treatment of post-traumatic bile leaks. We performed ERCP in 9 patients, 8 of which were performed within the first 24 hours after detection of a bile leak.

The success rate for therapeutic endoscopic intervention ranges from 90% to 100% [17-19]. Sugimoto et al. reported on healing of bile leaks in five of six patients after therapeutic ERCP [20]. Bajaj et al. reported a similar rate of success for therapeutic ERCP for the management of post-traumatic bile leaks [8 of 9 patients, (89%)] [21]. In our experience, ERCP as a primary therapeutic procedure was effective in 7 out of 9 patients (78%) with a bile leak. We were able to follow up 13 of the 16 patients (81.3%) who survived and observed that all 13 patients were doing well and had no liver-related complications at a median follow up of 7.5 months. As far as we can appreciate, non-operative treatment of biliary complications is associated with little or no long-term morbidity.

Similar to data from the literature, our results show that resolution of bile leaks occurred after a mean of 26 days (range 3-68 days), following endoscopic intervention [20,21]. The length of time for stenting varied from 3 to 8 weeks in previously published reports [19,22,23]. We remove stents after an interval of 12 to 24 weeks as a result of prolonged rehabilitation required following severe multitrauma. A cholangiogram before stent removal is necessary to confirm healing of bile duct injury. Our experience has shown that all biliary leaks healed, even those involving the main ductal system.

## Conclusions

In summary, patients with high-grade liver injury can develop complications necessitating a multidisciplinary approach. Liver complications should be expected in 30 to 70% and increase with grade of injury. Liver complications were not overwhelming and could be safely managed non-operatively. We advocate the use of CT in patients with clinical evidence of biliary complications and rebleeding, as a screening tool, before any invasive diagnostic and treatment procedures. Operative treatment and AE seem to be associated with an increased risk of biliary complications. A screening model should be developed for earlier diagnosis and treatment of complex hepatic-related complications in high-grade liver trauma victims.

## Competing interests

The authors declare that they have no competing interests.

## Authors' contributions

MB - main contributions to conception and design, acquisition of data, analysis and interpretation of data. SAG - participated in the design of the study, has made substantial contributions to conception and design, data collection and analysis. MF - participated in the design of the study and performed the statistical analysis. AB - participated in the design of the study, coordination and helped to prepare figures. GZ - participated in the design of the study and coordinated patients selection. AR - participated in the design of the study, coordination and helped to draft the manuscript. GA - participated in the design of the study and performed the statistical analysis, revising critically for important intellectual content. All authors read and approved the final manuscript.
